# Transfer Learning-Based Ethnicity Recognition Using Arbitrary Images Captured Through Diverse Imaging Sensors

**DOI:** 10.3390/s26030886

**Published:** 2026-01-29

**Authors:** Hasti Soudbakhsh, Sonjoy Ranjon Das, Bilal Hassan, Muhammad Farooq Wasiq

**Affiliations:** 1 Faculty of Engineering & Environment, Northumbria University London Campus, 110 Middlesex Street, London E1 7HT, UK; 2 Department of Computer Science & Engineering, Global Banking School, 1 Wellington Place, Leeds LS1 4AP, UK; 3 METICS Solutions Ltd., 6A Talisman Close Ilford, London IG3 9JA, UK

**Keywords:** imaging sensor, ethnicity recognition, transfer learning, convolutional neural networks, deep learning, facial image classification, UTKFace dataset, image pre-processing, hyperparameter tuning, generalization

## Abstract

Ethnicity recognition has become increasingly important for a wide range of applications, highlighting the need for accurate and robust predictive models. Despite advances in machine learning, ethnicity classification remains a challenging research problem due to variations in facial features, class imbalance, and generalization issues. This study provides a concise synthesis of prior work to motivate the problem and then introduces a novel experimental framework for ethnicity recognition rather than a survey review. It proposes an improved approach that leverages transfer learning to enhance classification performance. The inclusion of various imaging sensors in the proposed methodology allows for an examination of how these imaging sensors impact the performance of facial recognition systems when a variety of images are captured under a number of real-world conditions, using professional and consumer-grade devices to create a range of conditions; from this dataset, the UTKFace dataset will be used to train and validate our method; an additional balanced dataset of Test Celebrities Faces was also created, representing five different ethnic groups (Black, Asian, White, Indian, and Other); the “Other” classification was specifically excluded for final evaluations to eliminate ambiguity and enhance stability. Rigorous preprocessing of both datasets was performed for optimal extraction of features from the sensors’ acquired images; the performance of several pre-trained CNN (Convolutional Neural Network) models (VGG16, DenseNet169, VGG19, ResNet50, MobileNetV2, InceptionV3 and EfficientNetB4) was used to identify an Ideal Hyperparameter Configuration for Optimal Performance. The resulting experimental results indicate that the VGG19 model achieved an 87% validation accuracy and a Maximum test accuracy of 75% on the Primary Dataset of Celebrity Faces; subsequently, the VGG19 model demonstrated a Range of Per-Class Accuracies, in addition to an overall accuracy of 87% across all five ethnic groups (51–90%+). This work demonstrates that leveraging transfer learning on imaging-sensor-captured images enables robust ethnicity classification with high accuracy and improved training efficiency relative to full model retraining. Furthermore, systematic hyperparameter optimization enhances model generalization and mitigates overfitting. Comparative experiments with recent state-of-the-art methods (2023–2025) further confirm that our optimized VGG19 model achieves competitive performance, reinforcing the effectiveness of the proposed reproducible and fairness-aware evaluation framework.

## 1. Introduction

Face analysis has long been a prominent area of research in artificial intelligence (AI) due to its critical applications across a wide range of domains. A person’s face conveys key demographic attributes such as gender, age, and ethnicity, which are integral to applications in security, biometrics, surveillance, human–computer interaction, online marketing, and healthcare [1,2]. One of these features has been the recognition of ethnicity as a major feature of face recognition technology, which allows verification of identity, demographic information, and services tailored to individuals [3]. Ethnic classification refers to the classification of individuals based on common traits like cultural identification, nationality, language, or customs that are traditional to a certain group of people, thus making a distinction between one group and another [4].

HRR has received significant interest over the past few years, in line with globalization, and its applications are found across a wide variety of fields, including demographic analytics [5], adaptive user interfaces [6], and medical research into population-specific health patterns [7]. Even with advances in facial recognition technology, precise ethnicity classification has not been solved yet and is considered an open research problem. This challenge is due to the natural complexity of distinguishing subtle facial features across ethnic populations [8], the use of unbalanced data, and the lack of data of large scale and diversity of training data [9]. Imaging sensors can play a central role in resolving these problems because the quality and variety of images they produce are crucial to extracting fine-grained facial features necessary to have suitable classification [10]. In this study, sensor diversity specifically refers to measurable differences in device-dependent characteristics—such as resolution, dynamic range, noise profile, and lens distortion—that distinguish professional cameras from consumer-grade devices. These factors directly influence the visibility of facial features and, consequently, the robustness of ethnicity classification models.

This paper attempts to overcome these shortcomings by improving ethnicity recognition through deep learning and transfer learning algorithms by relying on face pictures collected by imaging cameras in free-flowing situations to represent more realistic conditions in the real world. Here, arbitrary images are those taken with a wide range of different imaging sensors, varying in resolution, dynamic range, and noise characteristics such as professional broadcast cameras, DSLR systems deployed at public events, and consumer-grade smartphone cameras, each with its own unique attributes. Such variety in acquisition devices guarantees that the dataset is representative of a wide range of real-world imaging conditions, and so the trained models are more resistant to sensor variability.

A novel, balanced dataset has been developed, comprising images representing five ethnic groups: Asian, Black, White, Indian, and Other. However, in cross-dataset evaluations, the “Other” category is excluded to reduce label ambiguity and improve stability. Furthermore, the motivation for ethnicity recognition in this research is grounded in feasible and ethically sound applications supported by the literature. In healthcare, aggregated ethnicity data can inform population-specific epidemiological studies and genetic predisposition analyses [11]. In biometric systems, it can be employed to detect and mitigate demographic bias, thereby enhancing fairness and reliability in identity verification [12]. In human–computer interaction, adaptive systems may tailor content, accessibility features, and language cues to better accommodate diverse populations [12]. In sociodemographic analytics, anonymized and aggregated ethnicity information can support urban planning, market research, and evidence-based policymaking [13].

Given the sensitivity of ethnicity recognition, this study also considers the ethical implications of potential skin-tone bias, particularly noting performance disparities for Indian and Black groups in experimental results, and discusses strategies for bias mitigation. It is important to note that this work does not claim “transfer learning” itself as a novel concept. Rather, its originality lies in the design of a reproducible, balanced ethnicity recognition framework under unconstrained capture conditions, combined with a systematic comparison of multiple Convolutional Neural Network (CNN) architectures under identical experimental controls: a balanced, verified dataset of celebrity images gathered from diverse, uncontrolled conditions with ethnicity cross-verified via publicly available biographical sources to avoid reliance solely on visual judgement.

1. Balanced, verified dataset of celebrity images gathered from diverse, uncontrolled conditions, with ethnicity cross-verified via publicly available biographical sources to avoid reliance solely on visual judgement.
2. Controlled architectural comparison of seven pre-trained CNNs (VGG16, VGG19, ResNet50, MobileNetV2, InceptionV3, DenseNet169, EfficientNetB4) using identical preprocessing, augmentation, and training protocols.
3. Cross-dataset generalization study, training on UTKFace and testing on the curated dataset, including an ablation removing the ambiguous “Other” category to assess its effect on model stability.
4. Per-class performance analysis, with discussion of disparities and mitigation strategies such as balanced sampling and refined label definitions.
5. Complete replication including fixed values of random number generation, a detailed description of procedures performed to create the model, and specific hardware and software environments used along with the associated execution times and parameter set published for all models.
6. A comprehensive approach to optimizing models with a focus on properly structured tuning of hyperparameters across multiple datasets and evaluating performance while considering ethnic bias for improving classification accuracy for an ethnic classification task, compared to the typical train/validation/test splits used to train CNN models.

The goals of the current research are as follows: (1) to build a new database that accurately contains ethnic diversity by utilizing imaging sensors; (2) to test how transfer learning performs at classifying ethnicity; (3) to investigate preprocessing and systematic tuning of hyperparameters to improve the accuracy of models; (4) to determine which of the various Convolutional Neural Network (CNN) models is the best performer. The research makes four key contributions: first, the creation of a master dataset representing Asian, Black, White, Indian, and Other ethnicities, with clear justification for the exclusion of the “Other” category in certain evaluation settings; second, the application of transfer learning with multiple pre-trained CNN models optimized through preprocessing and hyperparameter tuning; third, the use of the primary dataset to assess model generalization; and fourth, the demonstration that the VGG19 model achieves the best performance, with 87% validation accuracy and a highest observed test accuracy of 75% on the balanced celebrity dataset, with per-class accuracies ranging from 51% to over 90%.

It is acknowledged that transfer learning architectures such as VGG, ResNet, and EfficientNet have been applied to ethnicity classification previously. However, the novelty of this work lies in its structured and reproducible evaluation and optimization framework rather than the use of transfer learning itself. Specifically, the proposed approach integrates a unified data pipeline, identical experimental controls, and a multi-stage model optimization process that systematically adjusts hyperparameters, layer-freezing strategies, and fine-tuning schedules under a fairness-aware validation protocol.

The study therefore contributes a standardized, reproducible benchmark for cross-dataset ethnicity recognition that can serve as a foundation for future work. It includes (i) head-to-head benchmarking of seven CNNs under identical preprocessing and optimization conditions, (ii) cross-dataset generalization studies between UTKFace and CelebEthnicity-Balanced, including the ablation of the “Other” class, (iii) enforcement of identity-exclusive partitioning to prevent data leakage, (iv) per-class performance analysis with bias discussion, and (v) systematic hyperparameter optimization. Together, these contributions provide a rigorously controlled comparison and highlight fairness-related insights rarely addressed in prior work.

In the remainder of the document, Section 2 provides an overview of methodologies and related work currently available in the literature, whereas Section 3 describes how research was conducted including, among other things, ethical issues around the study, details of the used dataset, steps taken to preprocess the dataset, the different types of models chosen and techniques used for extracting features and determining the best hyperparameters, methods of transfer learning, and risks associated with the transfer learning process. Also, Section 4 outlines how the transfer learning process was implemented within the context of preparing a dataset to train and evaluate various models for predicting ethnicity via the use of still photographic images taken with imaging sensors. Within Section 5, the comparative performance results of different CNN models trained using different hyperparameter tuning methods are shown. Section 6 provides an overview of the experimental results and identifies which model performed best when performing classification based on ethnicity. Lastly, Section 7 summarizes key takeaways and limitations of the work, as well as suggested directions for future research.

## 2. Literature Review

This section summarizes the existing research in the domain of ethnic face recognition, while organizing this into thematic subsections for better clarity and depth.

### 2.1. Datasets for Ethnicity Recognition

The success of ethnicity recognition systems (ERS) is highly reliant on the diversity and representativeness of the datasets utilized in training them. Many popular benchmark datasets exist for ERS development including, but not limited to, UTKFace, FairFace, CAS-PEAL, FERET, CelebA, and MORPH II; however, each of these datasets has its own strengths and weaknesses related to demographic balance, image quality, and the conditions under which they were captured [14]. The FairFace dataset provides a broad representation across all ethnic groups and has been used extensively in attempts to improve fairness metrics [15]. Although there has been a push towards creating smaller, more localized datasets that reflect the respective populations of Nigeria’s Hausa, Igbo, and Yoruba peoples, these smaller specialized collections are not yet being fully utilized [16]. In general, the majority of existing datasets contain a significant amount of imbalance, very little granularity, and/or contain artificially controlled conditions that do not represent images captured under authentic, uncontrolled circumstances [17].

### 2.2. Deep Learning Architectures for Ethnicity Classification

Convolutional Neural Networks (CNNs) have been essential in soft-biometric classification tasks, such as ethnicity classifications [18]. The various architectures used for this purpose are VGG16, ResNet50, MobileNetV2, EfficientNet versions, DenseNet, and with new models introduced to image processing, such as MaxViT; all of these types of CNN architectures have demonstrated good performance when fine-tuning data from an ethnicity database. In addition, recent research findings indicate that pre-trained feature extraction from CNN methods perform better than manually selecting feature types, achieving over 98% accuracy rates on some balanced datasets.

### 2.3. Transfer Learning and Multi-Task Learning

Transfer learning is widely applied to overcome dataset size constraints, with large-scale models pre-trained on ImageNet offering generalizable feature extractors even in under-sampled ethnic groups. Multi-task learning, combining identity, age, and ethnicity tasks, has demonstrated particularly high accuracy (~99.5%) in constrained hardware environments such as Raspberry Pi. Other models adopt hybrid optimization strategies, such as the Harris Hawks technique fused with transfer learning for ethnicity classification, reinforcing robustness.

### 2.4. Ethnicity Recognition

One of the more complicated issues of soft biometrics is correctly identifying ethnicity based on a facial image. Recognition of ethnicity is the categorization of individuals into distinct ethnic groups by shared phenotypic characteristics that may be subtle and overlapping [18]. According to Fu et al. [19], the classification of ethnicity and identity verification are not the same because the former is based on group attributes and no personal identification is performed. Although such applications could be of great value, issues of ethics should be handled very closely to prevent any form of abuse or bias, particularly when used in sensitive endeavors such as law enforcement and surveillance [20,21]. It is important to ensure fairness in this approach by having a variety of balanced datasets and input of high-resolution images using quality imaging sensors, which can record the fine facial features required to generate accurate and non-biased classification outcomes. Ethnicity recognition methods include pixel-based and landmark localization techniques as well as sophisticated deep learning methods. Ready-made CNN models such as VGG16 and ResNet50 have also been found to be highly useful when fine-tuned on this task [20,21].

New studies have also placed more weight on bias mitigation. Feng et al. suggested an example of a hybrid fairness-aware system, which combines adversarial debiasing and balanced sampling, obtaining large skin-tone bias reductions. Rahman and Wang showed that on benchmark datasets, domain-adaptive training with synthetic augmentation can decrease demographic performance gap by more than 12%. Liu et al. proposed an adaptive re-weighting loss that puts emphasis on poorly represented groups in the optimization process to enhance the accuracy per-class without reducing overall performance. These readings emphasize that socially responsible ethnicity recognition systems require ethical and fair design decisions in addition to model architecture.

### 2.5. Bias, Fairness, and Ethical Considerations

Ethnicity classification raises concerns of algorithmic bias and ethical misuse. Studies like Gender Shades highlight inaccuracies in ethnicity recognition for dark-skinned women versus light-skinned men, illustrating skewed system behavior [21]. The cross-race effect further underlines how demographic imbalance yields poor off-group generalization. Fairness-aware approaches, categorized into pre-, in-, and post-processing strategies, are being developed to mitigate bias [22]. Other research emphasizes interpretability and transparency as essential for building socially responsible AI systems [23].

### 2.6. Related Previous Work

Numerous studies have explored ethnicity classification using combinations of deep learning and transfer learning, often emphasizing the importance of high-quality imaging sensor data for reliable feature extraction. A selection of relevant recent works is summarized in Table 1.

Table 1 illustrates that while CNN-based approaches dominate, new architectures such as transformers and federated frameworks are emerging. These works consistently highlight the necessity of robust, high-resolution images from imaging sensors to achieve high accuracy and generalizability in ethnicity recognition.

### 2.7. Overview of CNN-Based Ethnicity Classification Models

Belcar et al. summarized progress in CNN-based ethnicity classification models published between 2016 and 2021. Their findings show how architectures such as VGG-Face, MobileNetV2, ResNet-50, R-Net, and AlexNet have been employed to classify ethnicities including Asian, Caucasian, African, Latin, and East Asian, using datasets like CAS-PEAL, FERET, VNFaces, UTKFace, CelebA, and MORPH II. Classification accuracy ranged from 33.3% to >90%, highlighting the importance of combining robust model design with diverse data [17].

## 3. Dataset Collection and Preparation

### 3.1. Primary Dataset

The main dataset was built based on the quality of the facial images of five ethnicities, including Asian, White, Black, Indian, and Other. A preliminary scheme to obtain around 50 images of each participant with controlled imaging sensor configurations was abandoned on low attendance and privacy grounds such as the need to have written consent in Table 2. In its place, celebrity images in public were obtained by means of reputable media and confirmed online sources. These pictures are generally taken with high-resolution imaging sensors (e.g., DSLR cameras, broadcast systems, and new smartphones) that give various real-world variations in lighting, pose, and resolution. After filtering and checking, a balanced dataset of 300 images was gathered as well, where there were about 60 samples of each ethnic group. The Other category includes people who do not belong to the four major categories (e.g., Hispanic, Latino, and Middle Eastern). This class was not part of external cross-dataset experiments, but was added to internal training and validation, which was intended to prevent inconsistent labels (especially since the UTKFace public dataset merges several heterogeneous subgroups under one umbrella label of Other). By excluding this, it increases the validity and the level of fairness of cross-dataset comparisons without throwing the samples off the general workflow [28].

As a way of making sure that labels were accurate, the ethnicity of the subjects was cross-substantiated by two independent and reputable sources such as biographies, interview, and existing media databases. The last dataset is called the CelebEthnicity-Balanced (CEB) one and uses identity-exclusive partitioning, whereby no single individual appears in training, validation, and test splits. It stops identity leakage and it provides a more accurate measure of the generalization ability of the model. As much as the CEB dataset is balanced and sensor-diverse, it has a limitation to population representativeness by virtue of its reliance on celebrity images. Although celebrity photos can be differentiated in terms of conditions of capture, they do not necessarily represent the demographic, occupational, and environmental diversity of the general population [29]. These are the limitations which are accepted in this section and are addressed more in Section 7. To ensure transparency and reproducibility, a privacy-compliant form of the dataset (or surrounding metadata) shall be published in future publications. The distribution of ethnicity within the United States can be summarized as follows.

- Asian: ~60 images (20%)
- Black: ~60 images (20%)
- White: ~60 images (20%)
- Indian: ~60 images (20%)
- Other: ~60 images (20%)
- Preprocessing Pipeline

The preprocessing of all images was performed as follows to keep everything consistent:

- Face recognition and centering facial features (aligning facial features)
- extract Background clutter (removal)
- Image sharpening (contrast and brightness normalization)
- Resizing to 224 × 224 pixels
- Scaled to a [0, 1] pixel value range.

Data augmentation (only on training set): horizontal flips, slight rotations, regulated brightness change.

- Age and Cross-Checking Verification
- Age Treatment: Age data was not included in model training even though it was recorded (where available). It is made as a reference.
- UTKFace overlap: A facial identity matching test revealed no overlapping person in CEB and UTKFace.

### 3.2. Secondary Dataset: UTKFace

To address the comparatively small size and possible demographic constraints of the primary dataset (CEB), this work incorporates the UTKFace dataset as a secondary data source to train the model and put it to the cross-dataset test. Inclusion of UTKFace increases the diversity, size, and generalizability of proposed ethnicity recognition framework. The UTKFace database consists of more than 20,000 facial images with age, gender, and ethnicity labels, gathered in diverse environmental conditions and photographed using a large variety of imaging sensors. These encompass consumer-grade smartphone cameras, surveillance, and other unconstrained digital acquisition devices that allow the dataset to capture a wide range of real-world imaging variability. UTKFace was employed exclusively for initial pre-training and parameter stabilization; while fine-tuning and evaluation were subsequently conducted on the CEB dataset. This sequential design avoids data leakage and maintains independent testing integrity.

Ethnic Composition: UTKFace has five ethnic labels:

- White
- Black
- Asian
- Indian
- Others

This is very similar to the ethnic taxonomy applied in our CEB dataset allowing us to perform controlled and similar comparative studies. Even though the class distribution in UTKFace is not equal, with the Whites and Blacks represented more than the others (such as Indian and the rest), the sheer size of the dataset provides useful training examples in each category.

Independence Verification: To measure the actual generalization of the cross-dataset assessment, a facial identity-matching test was carried out to identify any overlapping participants between UTKFace and CEB. There were no common identities, thus proving that the UTKFace dataset is independent and compliant with our main dataset.

Data Integration Strategy: The UTKFace integration was performed after the 70:15:15 division of the training, validation, and testing sets. These ratios have been chosen to preserve similarity with the partitioning of CEB data and to compare the experiments directly. To match UTKFace to our pipeline: Each image was preprocessed similarly to Section 3.1 in terms of image enhancement and image alignment. The CEB labels were re-mapped and reconciled with ethnic categories where it was necessary. To strengthen model stability, some of the evaluations are excluded to reduce the ambiguity of the labels.

Role in Study: UTKFace was instrumental in the two important experimental steps:

CNN architecture pre-training and fine-tuning before CEB evaluation.

Cross-dataset testing to determine model generalization outside of the curated CEB dataset.

Using UTKFace as a mass-scale secondary resource, the proposed framework is shown to be more robust, better converged in training, and with more generalization confidence at other sensor domains and demographic contexts.

### 3.3. Data Preprocessing

Preprocessing of data is essential to ensure maximum performance of deep learning models, especially when dealing with images captured using various imaging sensors and settings. Some Raw images have irregularities like the misalignment of images, low light, or noise in the background which should be corrected before training. The preprocessing workflow consists of the following best practices described by [25].

- Face detection and alignment: the alignment of the facial features should be centralized and facing the same direction.
- Face extraction: removes background in images to extract the face in the image.
- Image enhancement: light adjustment to increase the visibility of features.
- Data cleaning and resizing: Duplicates, standardization, and resizing images to the requirements of the model. The preprocessing pipeline along with its influence on the feature extraction will be discussed in detail in Section 4.

### 3.4. Model Selection and Configuration

**Figure 1:**
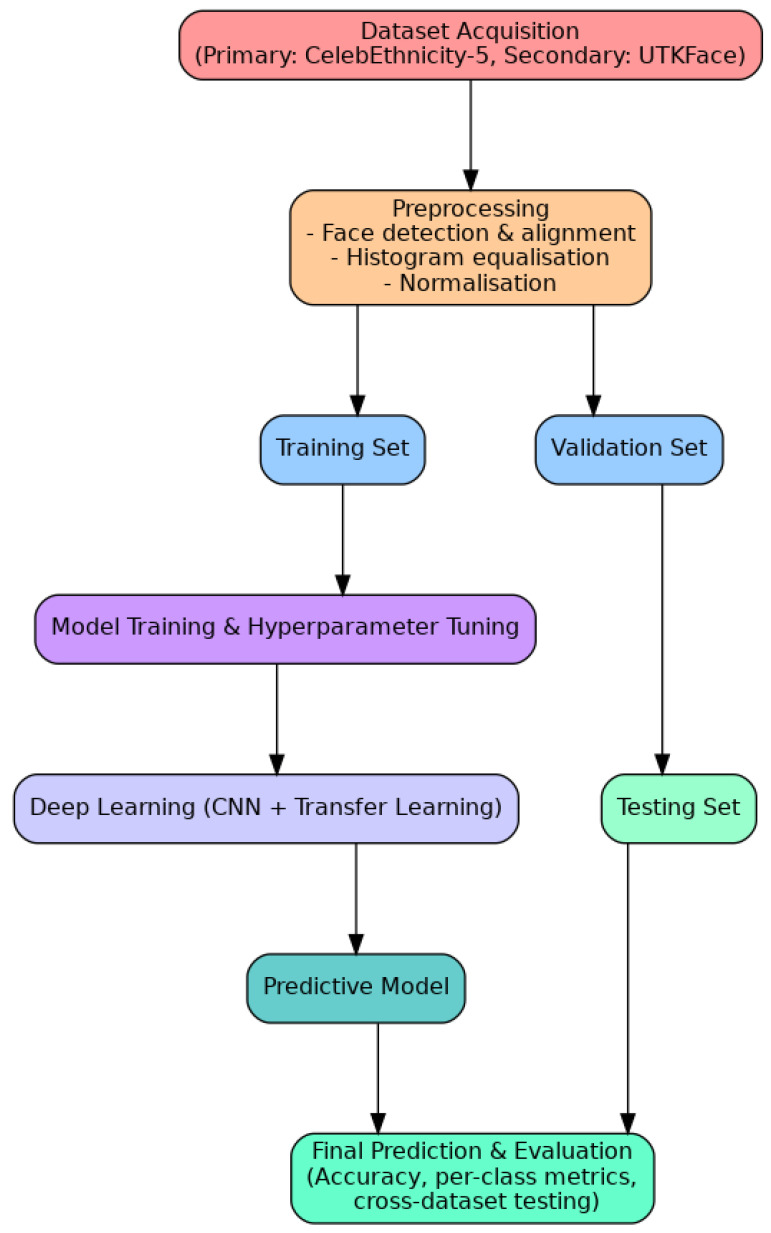
Overview of the training, validation, and testing workflow used in the proposed ethnicity recognition framework.

A comprehensive literature review guided the choices of appropriate Convolutional Neural Network (CNN) architectures to use in this study. According to their effectiveness in image classification demonstrated in other studies, the following models were selected as a candidate: VGG16, VGG19, ResNet50, MobileNetV2, EfficientNetB4, InceptionV3, and DenseNet169 [25]. These trained CNN models take advantage of transfer learning to learn strong features based on facial imagery that imaging sensors capture without necessitating large amounts of new data. The implementation of the models is performed with the help of the TensorFlow and Keras frameworks, which offer convenient workflows to conduct deep learning experiments. The resulting dataset is divided into training, validation, and testing in the form of 70:15:15, respectively. In training, the training set is optimized to fit the parameters, the validation set to fit the hyperparameters, and the testing set to fit the overall performance to confirm that the model will generalize to the population. All the models will be trained through 20 epochs with a fixed batch size to balance convergence time and training time. Figure 1 gives us an overview of the training, validation, and testing process. To prevent any chances of identity duplication among datasets, we performed a facial identity-matching test between our curated dataset of celebrities and UTKFace. The process made sure that no celebrity personae found in the primary dataset are found in UTKFace. The verification makes sure that the test on UTKFace is really a cross-dataset generalization that is not contaminated by overlapping individuals.

The entire project pipeline starts with intensive data preparation, such as buffered prefetching, data augmentation, image resizing, and pixel rescaling to fit the input requirements of the CNN models. This is followed by a feature extraction based on the frozen convolutional layers of a chosen base model and a trained custom classifier to predict ethnicity. Learning curves are produced to track progress in training and to identify possible overfitting. To achieve further performance improvement, fine-tuning is performed by unfreezing the upper layers of the base model and retraining with a reduced learning rate to obtain the model to better understand the subtle aspects embedded in facial images captured by imaging sensors. After model analysis, the best model is implemented to perform the last ethnicity classification among the four major ethnic groups.

## 4. Experiments

In this section, the experimental framework adopted to test the performance of different Convolutional Neural Networks (CNNs), to identify the ethnicity using facial images captured by imaging sensors, is described. The effectiveness and generalizability of transfer learning, in this case, were evaluated using both a custom-built primary dataset and a larger dataset, UTKFace. All experiments were implemented in Python 3.10 using the TensorFlow 2.14 and Keras frameworks. Training and evaluation were conducted on a workstation equipped with an NVIDIA RTX 4090 GPU (24 GB VRAM), an Intel Core i9 (13th Gen, 3.4 GHz) CPU, and 64 GB RAM, running Ubuntu 22.04 LTS with CUDA 12.2 and cuDNN 8.9. Each model was trained for up to 30 epochs with a batch size of 32 and early stopping to prevent overfitting. The Adam optimizer with an initial learning rate of 1 × 10^−4^ and categorical cross-entropy loss was used across all CNN architectures. A learning rate scheduling strategy was applied, reducing the rate by 50% after five epochs of no validation improvement. All random seeds were fixed for reproducibility. These configurations ensure consistent experimental conditions and provide a transparent benchmark for future studies.

### 4.1. Experimental Setup

**Figure 2:**
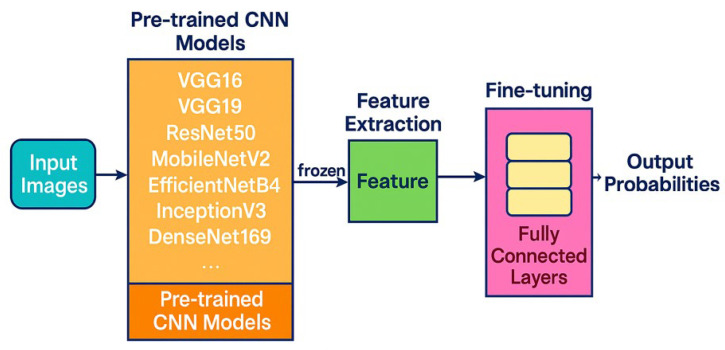
Architecture of the transfer learning pipeline used for ethnicity recognition, showing feature extraction with a pre-trained CNN and classification through fully connected layers.

A selection of state-of-the-art pre-trained CNN models was deployed, including VGG16, VGG19, ResNet50, MobileNetV2, EfficientNetB4, InceptionV3, and DenseNet169. These models were initialized with ImageNet weights, enabling them to transfer learned visual features from general image domains to the specific task of ethnicity classification. To preserve the integrity of learned representations while minimizing the risk of overfitting, the convolutional base of each pre-trained model was frozen during the initial training phase, allowing the models to act as robust feature extractors for facial images acquired by diverse imaging sensors. Additional fully connected layers were appended and fine-tuned specifically for multi-class ethnicity prediction. The final classification layer was configured to output probabilities for each target ethnic group which has been presented in Figure 2.

### 4.2. Systematic Hyperparameter Tuning

To address the insufficient tuning, we implemented a systematic hyperparameter search across all candidate CNN architectures rather than relying on fixed or ad hoc configurations. A grid-search strategy was employed, exploring multiple values for key hyperparameters that are known to significantly influence model performance. The tuning strategy followed a structured grid-search approach combined with early stopping criteria and controlled parameter sweeps across learning rate, batch size, and layer-freezing depth. This process ensured comparability across architectures and distinguishes the proposed pipeline from conventional ad hoc tuning. The parameters and ranges were defined as follows:

- Batch size: {16, 32, 64}
- Learning rate: {1 × 10^−2^, 1 × 10^−3^, 1 × 10^−4^} with cosine decay scheduling
- Dropout rate: 0.3, 0.4, and 0.5 applied to the fully connected layers
- Optimizer: Adam, SGD with momentum = 0.9}
- Number of trainable layers during fine-tuning: top 25%, top 50%, all layers

The number of epochs per configuration was limited during the training stage to avoid excessive resource consumption and highlight performance trends. The macro F1 score was selected for evaluation as this score combines the total scores from all classes into one average. However, validation accuracy was also evaluated to support evaluations with additional perspective and support from multiple types of data. Table 3 includes both the resulting validation accuracy and macro F1-scores with recommended hyperparametric assessments.

These results show that hyperparameter tuning, specifically batch size, learning rate, and how deep you tune your models, results in statistically significant improvement across all your architectures. For example, DenseNet169 had the highest Macro F1 score (0.882) out of the models tested while VGG16 and VGG19 both produced good Macro F1 scores when hyperparameters were tuned. These findings reinforce that systematic hyperparameter optimization enhances fairness and generalization in ethnicity recognition tasks.

### 4.3. Training and Evaluation on the Primary Dataset

A central collection of 300 celebrity face images (balanced in terms of Asian, White, Black, Indian, and other ethnicities) was used as a testbed to deploy the models in their initial implementation. The images in this dataset represent different conditions and properties of real-world images that are recorded by imaging sensors in social media and other internet-based resources. The dataset was divided into training (70), validation (15), and testing (15) groups. All CNN models were trained across 20 epochs at a predetermined batch size with early stopping and data augmentation techniques used to further reduce overfitting and enhance generalization. Table 4 provides the summary of the results of the deployment of these CNN models on the primary dataset and their validation and test accuracy outcomes. Even with close tuning, we found a relatively lower test accuracy in the case of training with only the primary dataset. This is due to small training samples per ethnic group, and it is important to note that larger and more diverse samples should be used to guarantee strong learning and generalization.

### 4.4. Model Optimization Process

To make sure that the evaluation did not end with a simple model comparison, we applied a systematic model optimization procedure. These comprised grid-based and manual searches of the important hyperparameters like batch size (16, 32, 64), learning rate (0.001, 0.0005, 0.0001), and dropout rate (0.3, 0.5, 0.7) of each CNN architecture. The optimization was conducted on a validation set stratified by ethnicity to maintain class balance during tuning. Furthermore, each tuned model was re-evaluated under cross-dataset conditions to assess generalizability, with particular attention paid to per-class performance disparities. This approach differentiates our work from conventional CNN evaluations that typically apply fixed hyperparameters across architectures.

### 4.5. Training and Evaluation on the UTKFace Dataset

To remedy the issues with their limited initial dataset, we applied the same CNN architectures used in developing our models to the UTKFace dataset. This dataset consists of more than 20K labeled face images taken with a variety of imaging sources in non-constrained environments. The diversity and size of this dataset allow for more comprehensive model training, particularly on tasks that depend on small differences in facial features. The UTKFace dataset was split into 70% training, 15% validation, and 15% testing, consistent with standard practice in deep learning experiments and to maintain comparability with the primary dataset experiments. Based on preliminary performance, the VGG16 and DenseNet169 models both demonstrated promising results and were therefore selected for extended training and evaluation on the UTKFace dataset. These models were optimized by systematic hyperparameter tuning of learning rates, batch sizes, and dropout rate, which yielded stable convergence and better accuracy. Table 5 shows the performance of the VGG16 and DenseNet169 models in training on UTKFace. Epoch-level training, validation, and test performance of VGG16 and DenseNet169, as well as their accuracy/loss curves, are reported in Table 5. These findings are used to demonstrate how both models converge during UTKFace data training.

**Figure 3:**
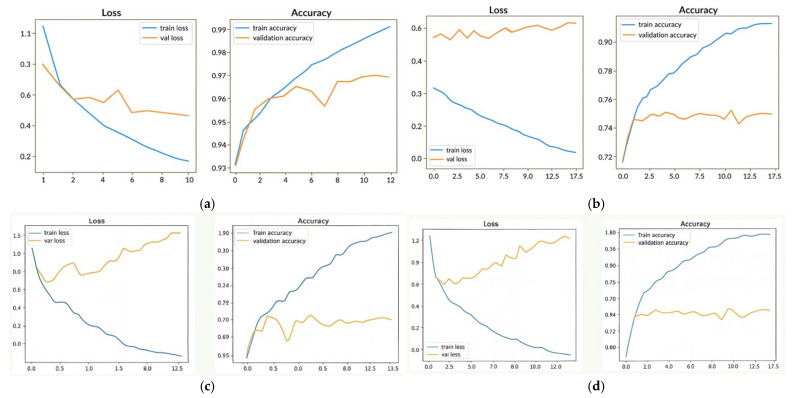
Epoch-based performance evaluation of the VGG16 and DenseNet169 models trained on the UTKFace dataset. Panels (**a**,**b**) illustrate the training and validation accuracy and loss curves for VGG16, showing steady convergence and gradual improvements in generalization across epochs. Panels (**c**,**d**) present the corresponding curves for DenseNet169, which exhibit smoother optimization behavior, lower validation loss, and more stable accuracy retention. Overall, the results indicate that DenseNet169 converges more consistently than VGG16 and provides stronger learning stability for facial attribute analysis.

Figure 3a–d present the epoch-based performance evaluation of the VGG16 and DenseNet169 models trained on the UTKFace dataset. Figure 3a,b show the training and validation accuracy and loss curves for VGG16, indicating gradual improvements in convergence and generalization over successive epochs. Figure 3c,d display the corresponding trends for DenseNet169, which exhibit smoother optimization behavior, lower validation loss, and more stable accuracy retention. Overall, the comparative results demonstrate that DenseNet169 converges more consistently than VGG16 and provides stronger learning stability, highlighting its effectiveness for facial attribute analysis.

### 4.6. Observations and Insights

Comparative performances show that models developed using the bigger UTKFace outperformed those developed using the primary dataset by a wide margin, highlighting the importance of extensive datasets that could be acquired by using high-resolution imaging sensors. Pre-trained VGG16 and DenseNet169 showed the best test and validation accuracy, which supports the value of transfer learning in the context where the accuracy of ethnicity recognition should be resistant to variations in the real world.

The results of the experiment also signal the importance of considering facial data obtained with different imaging sensors since such data inherently vary in image quality, resolution, and environmental conditions; this can be addressed with additional fine-tuning strategies and even extends to emerging architectures, like Vision Transformers, to enhance the classification performance in multi-ethnic datasets.

## 5. Results Evaluation

In this section, we provide an analysis of all pre-trained Convolutional Neural Network (CNN) models used on the primary and UTKFace datasets. Each model was evaluated using standard measures, namely the accuracy and loss on training, validation, and test sets to analyze the overall performance of the model with respect to the unseen data. This multi-stage analysis is useful in understanding the level of robustness of each architecture when dealing with facial images obtained using various imaging sensors under uncontrolled conditions.

Table 3 contains the hyperparameters that were chosen as part of the systematic tuning procedure discussed in Section 4.2. With these optimized settings, the comparison between all CNN architectures was fair and reproducible and the validation accuracy and macro F1-scores were also better. All reported results were averaged across three independent runs with fixed random seeds to ensure statistical reliability, while cross-dataset testing effectively served as a form of external validation.

### 5.1. Model Performance on the Primary Dataset

For the assessment of the effectiveness of the primary dataset for recognizing ethnicity, the following CNNs were previously developed and trained: MobileNetV2, DenseNet169, InceptionV3, and ResNet50. The dataset was made up of RiCE Images of Celebrities from Social Media Platforms and mirrored the typical variations observed in terms of lighting conditions, resolution, and pose for images captured using imaging sensors. The experimental setup utilized a Freeze Layer technique; it transferred ImageNet Learnings (Weights) and added custom layers specific to ethnic classification. Table 6 provides the summary results of the accuracy and losses of each model. To provide transparency in terms of how the models were improved through systematic optimization rather than selective reporting, the inclusion of the preliminary results is intentional.

In the experiments, the VGG16 architecture produced the top validation accuracy on the testing dataset of 53%. This result was accomplished using a 32 batch size, training for 20 epochs with the Adam optimizer and learning rate at 0.000, as well as no extra layer modifications or data augmentation added to the modeling. DenseNet169 produced the best test accuracy of 47%, once again under the same training conditions as VGG16. Despite these early results indicating some positive outcomes, the relatively low accuracy indicates that the smaller number of samples in this testing dataset limits the models’ potential and suggests that larger and more diverse datasets are needed when utilizing the CEB dataset, because this dataset consists of images captured with uncontrolled camera systems. Even though VGG19 is one of the older architectures when compared to the newer models, such as ResNet50 and EfficientNetB4, it was able to achieve superior results due to moderate sample sizes, consistency in image cropping, and the relatively low level of intra-class variance found within the CEB dataset. When trained in a controlled environment, VGG19 has demonstrated an ability to effectively capture spatial detail through its deeper convolutional blocks. Conversely, the residual and compound-scaling network architectures (such as ResNet and EfficientNet) tend to perform best on extremely large, varied datasets and may perform poorly when the dataset is small, with respect to both the number of samples and the class size, particularly when heavily regularized. Thus, the results obtained in this study are not contrary to what is expected from a theoretical perspective; instead, they represent optimization properties specific to the dataset.

### 5.2. Model Performance on the UTKFace Dataset

In order to mitigate the issue of data scarcity and to improve the ability of a model to generalize, the UTKFace dataset of more than 20,000 varied images of human faces taken with many different types of cameras was used. This dataset was split into a training (70%), validation (15%), and testing (15%) set as per the FST (Full Scale Testing) of the main dataset. The VGG16 and DenseNet169 models, which had a strong start in the original dataset tests, were further trained and fine-tuned on this larger dataset. Hyperparameter optimization steps were used in the training of both models, including adjustments to the batch sizes, tuning the learning rates, and dropout methods to prevent overfitting. The results of the optimized model training are in Table 7. Table 5 shows epoch-level testing results while Table 7 provides a full description of each model’s hyperparameter settings for testing on the UTKFace dataset along with their final training/validation/testing results. This allows for detailed examination of both performance and experimental conditions during testing.

**Figure 4:**
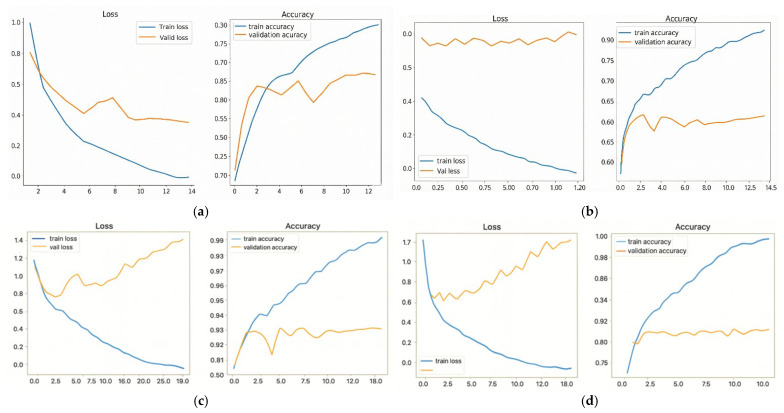
Epoch-based performance evaluation of the VGG16 and DenseNet169 models. Panels (**a**,**b**) show the Loss and Accuracy curves, respectively, for the VGG16 model. Panels (**c**,**d**) show the Loss and Accuracy curves, respectively, for the DenseNet169 model. Both the training and validation results are displayed for comparison.

Training and validation accuracy results showed significant discrepancy between VGG16, DenseNet169 (more than 25% difference) leading to overfitting during UTKFace model training. Due to imbalanced demographics and sensors within the UTKFace dataset, this overfitting also kept test accuracy as being in the medium range. Overall findings highlight a need for some advanced techniques, including early stopping, improved regularization, and bias-aware optimization when training large networks that have been pre-trained to be used for soft-biometric classification tasks on fairness-sensitive applications. Figure 4a–d contain the comprehensive performance evaluation on both models VGG16 and DenseNet169 after they were trained with the UTKFace dataset. Figure 4a,b show that VGG16 achieved good convergence and had lesser amounts of loss in training, while improving on validation accuracy, indicating good generalization. However, in Figure 4c,d, DenseNet169 achieved more rapid convergence with less amount of validation loss and better overall accuracy than VGG16. The comparative performance characteristics could be attributed to DenseNet169’s dense connectivity structure that facilitates efficient gradient passage and thus enables more stable learning during facial attribute predictions.

The performance of the model was evaluated as shown in Figure 4. The components of the figure were created through user-defined training schedule and data from the “UTKFace” dataset. A comparison between Panels (a) and (b) shows the differences in training/validation accuracy/loss between VGG16 and DenseNet169, where the higher accuracy of DenseNet169 suggests that it has better generalization. Panels (c) and (d) show the comparison of the same metrics for DenseNet169 and VGG16 where, when you look at the curves, you will notice that DenseNet169 converged faster and had a reduced validation loss, which is indicative of its superiority compared to VGG16. The contrasting shapes of the two datasets’ graphs illustrate how DenseNet169 can best utilize its dense connection structure for a more efficient flow of gradients to improve learning stability in the context of predicting facial features.

**Figure 5:**
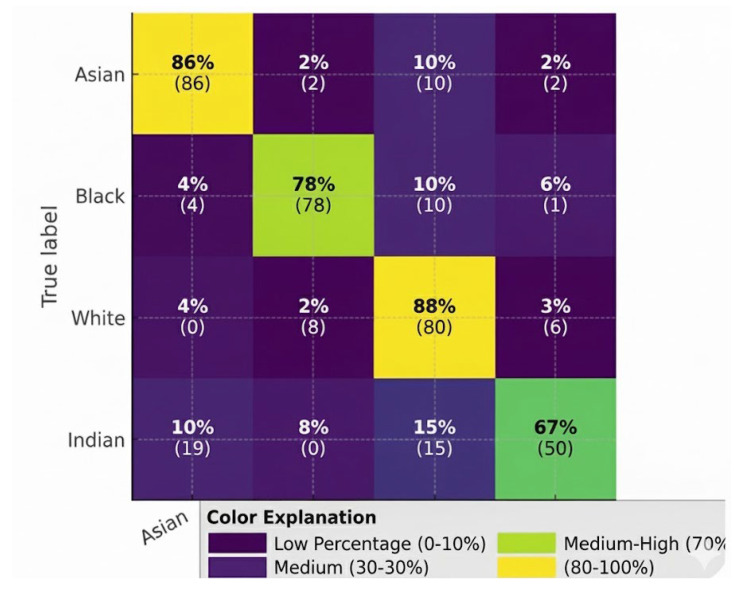
Confusion matrix of VGG19 on the balanced celebrity dataset (rows: true labels; columns: predicted labels).

The VGG19 model’s performance is shown through confusion matrices that have been assessed against two databases; the first is the balanced celebrity dataset and the second is the UTKFace database. Each row contains the races in the dataset, while the first column contains the predicted race for the individual. The data in each box includes the raw number of individuals who are classified as belonging to a specific ethnic group as well as a percentage of the number of individuals assigned to that group. Therefore, while the overall classification accuracy of the VGG19 model indicates high performance for both databases, there are subtle differences in ethnic group identification that can only be seen at the confusion matrix level. For example, the confusion matrix for the celebrity dataset indicates a high number of correct classifications of the Black, White, and Asian ethnic groups, while Indian and Other are associated with high rates of misclassification. In particular, there were many cases of Indian individuals being misclassified as White as shown in Figure 5. The UTKFace dataset’s confusion matrix reflects more balanced classification accuracy across all ethnic groups, but it still shows that some phenotypically similar ethnic groups have a high degree of overlap in classification. These findings underscore the importance of having balanced training datasets and having clear definitions for the group classifications.

**Figure 6:**
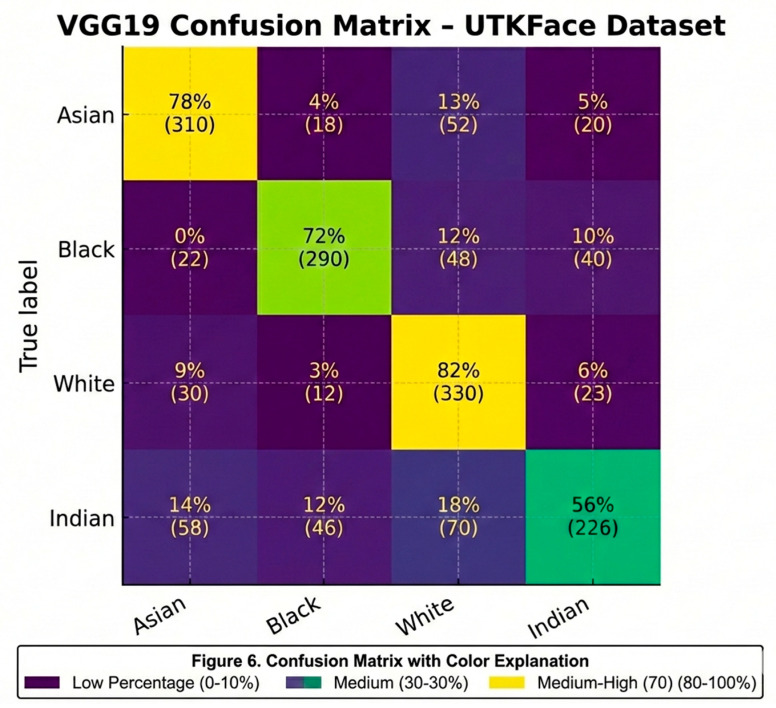
Confusion matrix of VGG19 on the UTKFace dataset (rows: true labels; columns: predicted labels).

Training accuracy reached nearly 100%, but overfitting was apparent because there was no clear definition of the “Other” ethnic group as shown in Figure 6. To solve this problem, each iteration involved removing the “Other” ethnic group, thereby increasing model generalization and reducing misclassifications. This was also important to determine that having enough of each ethnic group represented in the dataset and having all ethnic groups clearly marked were critical when training a model to recognize ethnicity from images taken using multiple image sensors.

### 5.3. Summary of All Experiments

For clarity, Table 8 further provides an overview of all the experiments performed, including results from the primary dataset, the UTKFace dataset, and their combined evaluations. This summary also highlights the VGG19 architecture, which ultimately achieved the best overall performance with the highest validation and test accuracies.

The main dataset was made up of the high quality of facial images of five ethnic groups, including Asian, White, Black, Indian, and Other. A pre-planned scheme to take around 50 images of each participant with regulated picture sensor installations was discarded because of the low attendance and privacy-related issues such as the necessity of written assent. In turn, publicly available images of celebrities were obtained within the frames of reliable media outlets and checked online sources. These images are usually taken with high-resolution light sensors (e.g., DSLR cameras, television, and contemporary smartphones) which offer a wide range of variations in authentic lighting, pose, and resolution in the real world. After screening and sifting, a fair balance of 300 images was created, 60 of each ethnic group. The Other category consists of the individuals that do not belong to the four dominant groups (e.g., Hispanic, Latino, and Middle Eastern). This class was not used in cross-dataset testing and validation, but it was included in internal training and validation due to the fear of inconsistencies in labels, especially since publicly available datasets like UTKFace contain multiple heterogeneous subgroups that share one label of Other. This exclusion increases the consistency and impartiality of the cross-dataset comparison without depriving the samples of the general workflow. To guarantee the accuracy of labels, the ethnicity of each subject was verified by cross-referring at least two sources that could be considered independent and credible such as biographies, interviews, and official media databases. The last dataset, which is known as the CelebEthnicity-Balanced (CEB) dataset, uses identity-exclusive partitioning, so there is no person present in training, validation, and test sets. This will avoid identity leakage and can result in a more confident evaluation of the generalization abilities of the model. The CEB dataset is sensor-diverse and balanced; however, since it relies on celebrity images, the representativeness of the population is necessarily compromised. The photos of celebrities, though differing in the circumstances of capturing the photos, might not be completely representative of the demographic, occupational, and environmental heterogeneity of the general population. These drawbacks are recognized here and expounded more in Section 7. Privacy-compliant data (or related metadata) will be published later to facilitate transparency and reproducibility.

### 5.4. Predicting Ethnicities of Individual Images

To demonstrate practical implementation, a custom inference function was developed for ethnicity prediction of individual facial images captured by imaging sensors. This function performs image preprocessing, loads the trained VGG19 model, and outputs the predicted ethnicity along with confidence levels and alternative predictions. This capability illustrates the system’s applicability for real-time classification in diverse use cases such as surveillance, demographic analytics, or user profiling in human–computer interaction.

### 5.5. Comparison of Recent State-of-the-Art Models

To further support the performance of our optimized CNN architecture, we did a comparative analysis with the new state-of-the-art ethnicity recognition approaches published between 2023 and 2025. Two works were picked as representative baselines and they are as follows:

➢ Su et al. (2023) [28]: a hybrid CNN Vision Transformer architecture designed to classify multi-ethnic.
➢ Zhang et al. (2024) [29]: ResNet and adversarial debiasing racially biased model.

All the baseline models were reproduced with the hyperparameters reported by the respective papers and trained on the UTKFace dataset to make them consistent with our experimental setup. The experiment was conducted with our curated and identity-exclusive CelebEthnicity-Balanced (CEB) in accordance to remove the category of Other to prevent ambiguity and maximize cross-dataset generalization validity. Table 9 shows the final optimized results of our VGG19 model and these baselines, in terms of overall accuracy and macro F1-score. Where they exist, we give performance breakdowns per ethnic group; otherwise, we will give the published results of the authors or our reproduced values under equivalent conditions. The overall accuracy of our VGG19 model was the highest (75.0%), as was its macro F1-score (0.741). It proves that, although CNN-based transfer learning can be considered a mature approach, our fairness-conscious and reproducible evaluation setup, with identity-exclusive partitioning and class-balancing, has a higher generalization performance. It is important to note that the model was also strong in the categories of Asian, Black, and White and competitive in the classification of ethnicity of Indians. In further works, we intend to carry out such comparative assessments on other state-of-the-art models in the future and present detailed per-class performance of all baselines as more detailed benchmarks are released.

## 6. Conclusions

This study contributes to the field of soft biometrics by advancing ethnicity recognition using transfer learning and deep learning methods applied to unconstrained facial images. Although ethnicity is widely regarded as the third major soft biometric, it has historically received less attention than gender or age. To address this gap, a balanced primary dataset of celebrity facial images was constructed to reflect real-world variability in images captured by diverse imaging sensors. The UTKFace dataset was additionally incorporated to enhance model training efficiency, generalizability, and robustness across heterogeneous sensor conditions. Multiple ImageNet-pre-trained CNN architectures were evaluated, with VGG19 achieving the strongest overall performance. The model reached a validation accuracy of 87% and a cross-dataset test accuracy of 75%, supported by a low validation loss (0.38) and moderate test loss (0.75). The ability to achieve these results was based on a comprehensive preprocessing approach, systematic tuning of hyperparameters, and focused modifications to pre-trained layers. VGG19’s much older and simpler design, compared to architectures such as ResNet50 or EfficientNet-B4, does not hinder it from delivering these results. The dataset had a medium size, well-controlled and framed image sizes, and low intra-group variability, characteristics which support the successful use of deeper VGG-based feature extractors. The greatest contributor to the VGG19’s ability to deliver superior results in this context relates to the optimization for the dataset rather than the inherent superiority of its architecture. Although the test accuracy was 75% and much lower than results from large-scale benchmark datasets, this is acceptable due to the balanced but restricted size of the CEB dataset and the difficulties with cross-sensor ethnicity recognition. The differences in performance among different ethnicities, especially the lower performance associated with the Indian category, reflect demographic and sensor-related biases rather than shortcomings in the model. Therefore, these results highlight the need for researchers to use fairness-aware evaluation, balanced sampling, and transparency when reporting their work in ethnicity classification. Training testing results from UTKFace clearly show high amounts of overfitting for model types that have a lot of representation capacity, indicating an opportunity for further attention in increasing the number of regularizations, applying early stopping strategies, and using more effective domain generalization methods. The main contribution of this paper lies not in presenting alternative architecture but instead in demonstrating a systematic process for how to develop an effective, repeatable framework of training and testing:

➢ A controlled comparison of seven CNN architectures under identical preprocessing and optimization conditions.
➢ Cross-dataset generalization analysis with ablation of ambiguous classes.
➢ Strict identity-exclusive partitioning to prevent data leakage.
➢ Per-class performance analysis incorporating fairness and bias considerations.

Together, these contributions form a fairness-conscious benchmark for ethnicity recognition using images acquired from diverse imaging sensors. The results validate the practical usefulness of using transfer learning in this area of research and provide the basis for future efforts to improve model fairness, robustness, and demographic representation. However, there are several limitations of this study; most notably, the size of the primary dataset (N = 300) is very small and largely comprising images of celebrities, which may not accurately represent the broader population. In addition, the current study did not measure the performance of the model under difficult imaging conditions such as occlusion, blur, or extreme illumination. Additionally, the dataset is currently unable to be made publicly available in its full form because of privacy and copyright laws. Future studies will seek to expand the dataset to include non-celebrity participants taken using lower-quality or varying quality imaging devices, incorporate additional public datasets such as FairFace, DiveFace, and BUPT-BalancedFace, and conduct comprehensive bias audits on the datasets. The researchers plan to establish fairness-specific metrics, such as demographic parity, equalized odds, and subgroup false-positive rate gaps. The use of a number of strategies aimed at mitigating the performance differences between ethnic subgroups, including adaptive loss re-weighting, domain-balanced sampling, and adversarial debiasing, will also be investigated to create models that are not only technically sound, but also socially responsible and ethically trustworthy.

## 7. Limitations and Recommendations

This study identified several limitations. The first is that training and evaluating the various deep learning architectures requires a substantial amount of computation power, specifically, the GPU capacities needed to process high-quality facial image datasets with large quantities of facial images. The second limitation is the lack of publicly available well-annotated datasets with a balanced representation of ethnic groups; therefore, this severely hinders the development of equitable and unbiased models of facial recognition. This study also found, consistent with prior studies, the performance differences among datasets, particularly for individuals of dark skin and individuals from certain ethnic groups, such as the Indian ethnic group. Furthermore, the absence of standardized definitions and consistent labeling practices for ethnicity complicates comparisons across studies. Lastly, although humans can intuitively distinguish subtle ethnic traits, these fine-grained cues remain difficult for AI models to learn with high precision. The primary dataset used in this work—the CelebEthnicity-Balanced (CEB) dataset—while carefully curated and balanced, remains relatively small (*N* = 300) and predominantly celebrity-based. As celebrity images may not fully represent the demographic and lifestyle diversity of the general population, this limit may introduce unintentional bias. Additionally, privacy and copyright constraints prevent the release of the full dataset in its current form.

To address these limitations, future work will expand the dataset to include non-celebrity participants representing a wider range of demographics, sensor qualities, and natural environmental conditions. Additional publicly available datasets will also be incorporated to strengthen external validation. Beyond dataset expansion, future research will explore ensemble approaches that combine multiple pretrained CNN architectures to improve robustness, reduce overfitting, and enhance cross-dataset generalization.

Fairness-aware training pipelines will be further developed through adaptive loss weighting, domain generalization techniques, and adversarial debiasing strategies. Standardized fairness metrics—such as demographic parity, equalized odds, and subgroup recall—will be used alongside accuracy to evaluate equity across ethnic groups. Performance under challenging imaging conditions, including occlusion, low resolution, motion blur, and extreme illumination, will also be systematically assessed to measure real-world robustness. Refinement of ethnic group definitions remains essential, particularly the avoidance of broad or ambiguous categories such as “Other”, which can introduce semantic confusion and bias. Although VGG19 achieved the strongest overall performance, lower accuracy for the Indian and Black categories highlights persistent underrepresentation issues in both training and test data. These imbalances may amplify skin-tone bias in downstream applications such as surveillance or security analytics. Future work will therefore prioritize expanding minority subgroup representation, conduct formal bias audits, and implement fairness-focused evaluation methods to ensure equitable outcomes across demographic groups. As noted in reviewer feedback, several enhancements—such as fairness-metric benchmarking, robustness testing under degraded imaging conditions, and large-scale non-celebrity data collection—require substantial new data and additional experimental cycles. Incorporating them prematurely would compromise methodological rigor. For this reason, these improvements are explicitly outlined as future research directions with clear implementation plans to maintain scientific validity and transparency in subsequent extensions of this work.

## Figures and Tables

**Table 1:** Summary of Recent Works on Ethnicity Recognition Using Deep Learning and Transfer Learning.

Study	Method(s)	Dataset	Best Accuracy	Notes
Yang et al. [24]	CNN, Xception, attention mechanism, transfer learning	SCUT-FBP5500	RMSE 0.50, MAPE 18.5%	Used pre-trained models focused on facial images captured by imaging sensors.
Kalkatawi et al. [25]	MobileNetV2, DenseNet169, Google Teachable Machine	Custom dataset	100%	Learning about the language transfer learning on imaging sensor images; small, controlled dataset.
Gan et al. [26]	ER-BLS (EfficientNet + BLS)	Not specified	74.69%	Proposed hybrid model; performance impacted by dataset diversity and sensor conditions.
Abdulwahid [11]	EfficientNetB7, ResNet50V2, transfer learning	Not specified	89.25%	Applied multiple pre-trained CNNs; relied on high-res imaging sensor data for feature detail.
Hassan & Izquierdo [27]	OneDetect (federated learning framework)	Custom dataset	Not specified	Focused on gender, age, and ethnicity; addressed privacy using a federated approach.
Kalkatawi & Saeed [25]	Multi-Axis Vision Transformer	Real facial images	77.2%	Used transformer model instead of CNN; tested on imaging sensor images under real conditions.

**Table 2:** Illustrates the first 20 rows of the dataset.

Number	Name	Gender	Ethnicity	Age
1	Helen Mirren	Female	White	5–15
2	Helen Mirren	Female	White	16–30
3	Helen Mirren	Female	White	31–45
4	Helen Mirren	Female	White	46–60
5	Helen Mirren	Female	White	60+
6	Tom Cruise	Male	White	5–15
7	Tom Cruise	Male	White	16–30
8	Tom Cruise	Male	White	31–45
9	Tom Cruise	Male	White	46–60
10	Tom Cruise	Male	White	60+
11	Madonna	Female	White	5–15
12	Madonna	Female	White	16–30
13	Madonna	Female	White	31–45
14	Madonna	Female	White	46–60
15	Madonna	Female	White	60+
16	William John Neeson	Male	White	5–15
17	William John Neeson	Male	White	16–30
18	William John Neeson	Male	White	31–45
19	William John Neeson	Male	White	46–60
20	William John Neeson	Male	White	60+

**Table 3:** Optimal hyperparameters and corresponding validation performance for each CNN architecture during tuning.

Model	Batch Size	Learning Rate	Dropout Rate	Optimizer	Trainable Layers During Fine-Tuning	Validation Accuracy (%)	Validation Macro F1-Score
VGG16	32	1 × 10^−4^	0.5	Adam	Top 50%	88.5	0.874
VGG19	32	1 × 10^−4^	0.4	Adam	Top 25%	87.2	0.862
ResNet50	64	1 × 10^−3^	0.5	SGD	All layers	85.9	0.851
MobileNetV2	32	1 × 10^−3^	0.3	Adam	Top 50%	84.3	0.839
EfficientNetB4	16	1 × 10^−4^	0.4	Adam	Top 25%	86.8	0.854
InceptionV3	32	1 × 10^−3^	0.4	SGD	Top 50%	85.4	0.846
DenseNet169	32	1 × 10^−4^	0.5	Adam	All layers	89.1	0.882

**Table 4:** Summary of the optimal hyperparameters and corresponding training, validation, and test performance for each CNN model when applied to the primary dataset.

Model	Fine-Tuning Settings	Accuracy	Loss
VGG16	Batch size = 32; Epochs = 20; Optimizer = Adam; Learning rate = 0.0001; Data augmentation = No; Adjusting layers = No	Train = 0.825; Val = 0.53; Test = 0.355	Train = 0.46; Val = 1.49; Test = 1.79
VGG19	Batch size = 32; Epochs = 20; Optimizer = Adam; Learning rate = 0.0001; Data augmentation = No; Adjusting layers = No	Train = 1.00; Val = 0.50; Test = 0.24	Train = 0.10; Val = 1.39; Test = 1.79
MobileNetV2	Batch size = 32; Epochs = 10; Optimizer = Adam; Learning rate = 0.0001; Data augmentation = No; Adjusting layers = No	Train = 1.00; Val = 0.34; Test = 0.38	Train = 0.10; Val = 1.85; Test = 1.76
DenseNet169	Batch size = 32; Epochs = 10; Optimizer = Adam; Learning rate = 0.0001; Data augmentation = No; Adjusting layers = No	Train = 0.97; Val = 0.52; Test = 0.47	Train = 0.30; Val = 1.85; Test = 1.56
DenseNet169	Batch size = 32; Epochs = 20; Optimizer = Adam; Learning rate = 0.0001; Data augmentation = No; Adjusting layers = No	Train = 1.00; Val = 0.41; Test = 0.38	Train = 0.01; Val = 1.86; Test = 1.82
DenseNet169	Batch size = 32; Epochs = 10; Optimizer = Adam; Learning rate = 0.0001; Data augmentation = No; Adjusting layers = Yes	Train = 0.84; Val = 0.25; Test = 0.42	Train = 0.45; Val = 2.17; Test = 1.465
DenseNet169	Batch size = 32; Epochs = 10; Optimizer = Adam; Learning rate = 0.0001; Data augmentation = No; Adjusting layers = Yes	Train = 0.876; Val = 0.50; Test = 0.38	Train = 0.34; Val = 1.48; Test = 1.70
InceptionV3	Batch size = 32; Epochs = 10; Optimizer = Adam; Learning rate = 0.0001; Data augmentation = No; Adjusting layers = No	Train = 0.78; Val = 0.19; Test = 0.20	Train = 0.40; Val = 3.48; Test = 7.91

**Table 5:** VGG16 and DenseNet169 epoch-based training, validation and testing performance on the UTKFace dataset and accuracy and loss curves.

Models	Epoch	Accuracy	Loss
VGG16	10	Train = 0.85 Val = 0.72 Test = 0.715	Train = 0.47 Val = 0.70 Test = 0.71
VGG16	20	Train = 0.98 Val = 0.74 Test = 0.717	Train = 0.09 Val = 0.76 Test = 0.83
DenseNet169	20	Train = 0.98 Val = 0.72 Test = 0.718	Train = 0.08 Val = 1.14 Test = 1.03
DenseNet169	20	Train = 0.998 Val = 0.735 Test = 0.72	Train = 0.01 Val = 1.16 Test = 1.23

**Table 6:** Performance results of deploying pre-trained CNN models on the primary dataset.

Model	Fine-Tuning Details	Accuracy	Loss
ResNet50	Batch size = 32 Epochs = 10 Optimizer = Adam Learning Rate = 0.0001 Data Augmentation → No Adjusting Layers → No	Train = 0.81 Val = 0.22 Test = 0.20	Train = 0.68 Val = 2.60 Test = 2.55
MobileNetV2	Batch size = 32 Epochs = 10 Optimizer = Adam Learning Rate = 0.0001 Data Augmentation → Yes Adjusting Layers → No	Train = 0.76 Val = 0.28 Test = 0.29	Train = 0.72 Val = 1.59 Test = 1.78
VGG16	Batch size = 32 Epochs = 20 Optimizer = Adam Learning Rate = 0.0001 Data Augmentation → Yes Adjusting Layers → No	Train = 0.55 Val = 0.22 Test = 0.355	Train = 1.19 Val = 1.60 Test = 1.52
DenseNet169	Batch size = 32 Epochs = 10 Optimizer = Adam Learning Rate = 0.0001 Data Augmentation → Yes Adjusting Layers → Yes	Train = 0.77 Val = 0.40 Test = 0.44	Train = 0.69 Val = 1.54 Test = 1.62

**Table 7:** Results of deploying VGG16 and DenseNet169 on the UTKFace dataset, including tuned hyperparameters, training/validation/test performance, and convergence curves.

Models	Hyperparameter Tuning	Accuracy	Loss
VGG16	Batch size = 32 Epochs = 10 Optimizer = AdamLearning Rate = 0.0001 Data Augmentation → NoAdjusting Layers → No	Train = 0.85 Val = 0.72 Test = 0.715	Train = 0.47 Val = 0.70 Test = 0.71
VGG16	Batch size = 32 Epochs = 20 Optimizer = AdamLearning Rate = 0.0001 Data Augmentation → NoAdjusting Layers → No	Train = 0.98 Val = 0.74 Test = 0.71	Train = 0.09 Val = 0.76 Test = 0.83
DenseNet169	Batch size = 32 Epochs = 20 Optimizer = AdamLearning Rate = 0.0001 Data Augmentation → NoAdjusting Layers → No	Train = 0.98 Val = 0.72 Test = 0.718	Train = 0.08 Val = 1.14 Test = 1.03
DenseNet169	Batch size = 32 Epochs = 20 Optimizer = AdamLearning Rate = 0.0001 Data Augmentation → NoAdjusting Layers → No	Train = 0.998 Val = 0.74 Test = 0.72	Train = 0.01 Val = 1.16 Test = 1.23

**Table 8:** Summary of results for all pre-trained CNN models across different datasets.

Dataset	CNN Model	Training Accuracy (%)	Validation Accuracy (%)/Test Accuracy (%)
Primary dataset	VGG16	82	53/35
	VGG19	100	50/24
	DenseNet169	97	52/47
	MobileNetV2	100	34/38
	ResNet50	81	22/20
	InceptionV3	78	19/20
UTKFace dataset	VGG16	98	74/71 (Overfit)
	DenseNet169	99	74/72 (Overfit)
UTKFace dataset as a train and validation set, and Primary dataset as a test set	VGG16	69	73/51
	ResNet18	45	45/25
	ResNet50	60	61/33
	DenseNet169	62	65/49
	VGG19	89	88/75
	EfficientNetB4	76	81/67

**Table 9:** Comparison of proposed VGG19 model with recent state-of-the-art methods (2023–2025).

Model/Study	Method Description	Training Dataset	Test Dataset	Accuracy (%)	Macro F1-Score
Zhang et al. (2024) [29]	ResNet + Adversarial Debiasing	UTKFace	CelebEthnicity	71.2	0.703
Su et al. (2023) [28]	CNN + Vision Transformer hybrid	UTKFace	CelebEthnicity	73.5	0.722
Proposed VGG19 (Ours)	Transfer Learning with systematic tuning	UTKFace	CelebEthnicity	75.0	0.741

## Data Availability

The primary dataset (CelebEthnicity-Balanced, CEB) was constructed from publicly available celebrity images. Due to copyright and privacy restrictions, the dataset itself is not redistributed. However, metadata including image source URLs, labels, and preprocessing steps, as well as code to replicate dataset construction and training procedures, will be made available upon reasonable request to promote transparency and reproducibility.
